# Five-year associations among dopamine D2-like receptor loss, cognitive decline, education, and self-reported leisure activities in healthy older adults

**DOI:** 10.1162/IMAG.a.1302

**Published:** 2026-07-21

**Authors:** Amos Pagin, Nina Karalija, Micael Andersson, Lars Nyberg, Lars Bäckman, Katrine Riklund, Ulman Lindenberger, Martin Lövdén

**Affiliations:** Department of Psychology, University of Gothenburg, Gothenburg, Sweden; Umeå Center for Functional Brain Imaging, Umeå University, Umeå, Sweden; Department of Medical and Translational Biology, Umeå University, Umeå, Sweden; Department of Diagnostics and Intervention, Umeå University, Umeå, Sweden; Aging Research Center, Karolinska Institute and Stockholm University, Solna, Sweden; Center for Lifespan Psychology, Max Planck Institute for Human Development, Berlin, Germany; Max Planck Centre for Computational Psychiatry and Ageing Research, London, United Kingdom

**Keywords:** dopamine, cognition, aging, education, leisure activities, cognitive reserve

## Abstract

Age-related loss of dopamine (DA) integrity has been linked to cognitive decline. Relatedly, education and leisure activity engagement have been highlighted as neurocognitive protective factors, but their associations with DA integrity remain poorly understood. Using Bayesian structural equation modeling, we analyzed longitudinal data from the Cognition, Brain, and Aging (COBRA) prospective cohort study with 181 older adults at baseline to examine correlations among DA D2-like receptor (DRD2) availability in the caudate and putamen, measured using [^11^C]raclopride positron emission tomography (PET), cognition (working memory, episodic memory, and perceptual speed), education, and self-reported physical, cognitive, and social leisure activity measures. Our research questions target whether (i) education or leisure activities are associated with baseline levels or 5-year changes in DRD2 availability; (ii) changes in leisure activities covary with DRD2 changes; and (iii) education or leisure activities moderate DRD2–cognition change–change correlations. Results showed declines in DRD2 availability in the caudate and putamen, with weak overall DRD2–cognition change–change correlations. For both baseline levels and changes in DRD2 availability, the associations with education and leisure activities were uniformly negligible or small and not strongly supported. Neither education nor leisure activities moderated DRD2–cognition change–change correlations.

## 1 Introduction

Aging is associated with group-average decline in several cognitive abilities, including memory, executive functions, and processing speed, but there are individual differences in these within-person changes (Raz et al., 2005; Rönnlund et al., 2005; Schaie, 1994). Relatedly, the neurotransmitter dopamine (DA) has been linked to cognitive functioning (Bäckman, Karlsson, et al., 2011; Bäckman et al., 2006; Cools & D’Esposito, 2011; Cropley et al., 2006; Egerton et al., 2009; Nyberg et al., 2016), and linear or accelerated losses in DA biomarkers occur from young-old to very old age (Antonini et al., 1993; Bäckman et al., 2006; Bannon & Whitty, 1997; Karalija et al., 2022; Ma et al., 1999; Rinne et al., 1990). Due to its dense dopaminergic innervation, research on the association between DA and aging has primarily emphasized the striatal complex, where evidence from both autopsy studies and in vivo molecular imaging studies using positron emission tomography (PET) or single-photon emission computed tomography (SPECT) has described age-related declines in DA D1 and D2 receptor densities at an estimated rate of 8%–14% per decade, and presented similar estimates for losses in DA transporter (DAT) densities (Karrer et al., 2017; Rieckmann et al., 2011). These observations have led researchers to propose that DA integrity may be an important brain correlate for age-related cognitive decline (Bäckman et al., 2006, 2010; Nevalainen et al., 2015).

In recent publications, we have demonstrated empirical support for correlated DA–cognition changes in aging using longitudinal data from the Cognition, Brain, and Aging (COBRA) prospective cohort study (Karalija et al., 2024; Lundgren et al., 2025; Nevalainen et al., 2015; Papenberg et al., 2025). In COBRA, healthy older adults (*n* = 181; age range: 64–68 years, *M* = 66.2 at baseline) underwent repeated PET imaging using [^11^C]raclopride to assess dopamine D2-like receptor (DRD2) availability, along with cognitive assessments of episodic memory, working memory, and perceptual speed. Initial analyses of 5-year decline rates of striatal DRD2 and cognition revealed weak overall DRD2–cognition change correlations in the full sample of returnees, but significant change correlations for a subsample of individuals with more extensive DRD2 decline (Karalija et al., 2024), as well as for working memory (Papenberg et al., 2025). In a recently completed analysis of the 10-year follow-up, we observed correlated 10-year declines in striatal DRD2 availability and a general cognition factor (*r* = .31, pD_>0_ > .95; Lundgren et al., 2025).

The evidence for a link between age-related declines in DA and cognitive performance invites further probing into questions concerning protective and risk factors for preservation of late-life DA integrity. To date, little is known about such factors. Related research on cognitive impairment has frequently highlighted educational attainment and late-life engagement in physical, cognitive, and social leisure activities as factors that may delay or attenuate cognitive decline (Andel et al., 2016; Lövdén et al., 2005; Stern et al., 2020; Yang et al., 2022; Yates et al., 2016), either through their contributions to a cognitive or brain reserve (Katzman et al., 1988; Stern, 2002, 2009; Stern et al., 2020), to brain maintenance (Nyberg et al., 2012), or through some other protective mechanism (for a critical review, see Lövdén et al., 2020). In recent years, the hypothesis that education slows brain aging has been challenged by several longitudinal studies (Fjell et al., 2025; Lövdén et al., 2023; Nyberg et al., 2021), but such studies have mainly examined structural MRI-derived measures of brain atrophy rather than age-related changes in neurotransmitter systems. Accordingly, a question of current interest is whether education or leisure activity engagement is associated with baseline levels or longitudinal changes in DA integrity. A closely related question is whether education moderates the DA–cognition change–change correlations, as would be consistent with cognitive reserve theory (Stern et al., 2020). Specifically, cognitive reserve theory predicts that individuals higher in cognitive reserve (e.g., highly educated individuals) are less cognitively affected by adverse brain changes compared with individuals lower in cognitive reserve (e.g., individuals with lower education). However, the relevance of education for DA–cognition associations in aging has not been examined longitudinally.

In terms of empirical evidence, studies have linked both acute and prolonged physical exercise to elevated DA concentrations in rodents (de Castro & Duncan, 1985; Hattori et al., 1994; Meeusen et al., 1997; Petzinger et al., 2007), and human PET studies examining healthy older adults have linked aerobic fitness and self-reported physical activity intensity to striatal DRD2 density (Jonasson et al., 2019; Köhncke et al., 2018). However, exercise intervention studies in both animals and humans have reported mixed findings on the effects of exercise on striatal DRD2 densities (Jonasson et al., 2019; Robertson et al., 2015; Wang et al., 2000). Similarly, cognitive training has been shown to induce increased cortical densities of DA D1 receptors (McNab et al., 2009) and enhanced striatal DA release as indicated by reduced DRD2 availability (Bäckman & Nyberg, 2013; Bäckman, Nyberg, et al., 2011). In the social domain, PET studies have linked striatal DRD2 availability to social status and perceived social support (Martinez et al., 2010). These findings suggest that physical, cognitive, and social leisure activities could be associated with DA functioning. With regard to education, only a few cross-sectional studies have examined links between educational attainment and DA functioning, describing mixed results: One SPECT study linked higher education to higher striatal DAT binding in older patients with Lewy body dementia (Lamotte et al., 2016), but a similar SPECT study found no association between education and striatal DAT binding in either older patients with Parkinson’s disease or age-matched healthy controls (Hoenig et al., 2023).

DA–cognition associations in aging, including examinations of potential protective factors for DA integrity, represent areas of strong research interest. To our knowledge, no large-scale longitudinal PET study has examined whether education or leisure activity engagement is associated with DRD2 decline, or whether these factors moderate DRD2–cognition change associations. We here present results from a longitudinal investigation addressing these questions using data from the COBRA study (Nevalainen et al., 2015). In line with previous research, our attention is focused on the striatal complex. Moreover, we examine whether education or physical, cognitive, or social leisure activity engagement moderates DRD2–cognition change–change correlations, focusing on episodic memory, working memory, and perceptual speed. To ensure robustness and computational feasibility of our large set of Bayesian models, we focus our analyses on the first two of the three waves of COBRA data.

## 2 Method

We refer to previous publications for a detailed description of the overall design, statistical power analyses, recruitment procedure, imaging protocols, cognitive tests, and questionnaires used in COBRA (Nevalainen et al., 2015). We report here only the methodological details of direct relevance to the current study.

### 2.1 Sample

In COBRA, 181 healthy older adults (64–68 years; *M* = 66.2, *SD* = 1.2; 81 women) were randomly selected from the population register of Umeå in northern Sweden. The age range was chosen to reduce age- and cohort-related heterogeneity and to minimize attrition due to morbidity and mortality over the planned 10-year follow-up period. Exclusion criteria were neurological and psychiatric disorders, epilepsy, previous brain trauma, intellectual disability, a Mini-Mental State Examination (MMSE) score below 27, structural brain abnormalities (inspection performed by neuroradiologists), cancer, diabetes, severe auditory and visual impairments, claustrophobia, and metal implants. Of the original sample, 129 participants returned for the 5-year (T2) follow-up (69–73 years; *M* = 71.2, *SD* = 1.2; 60 women). Attrition analyses reported in Karalija et al. (2022) indicated that longitudinal selectivity was modest (0.10–0.16 SDs): Compared with returnees, dropouts were more likely to be retired (84.6% vs. 66.7%, *p* = .015), showed lower baseline cognitive performance (vocabulary; digit-symbol coding; MMSE), reported lower intellectual activity levels, had higher cardiovascular disease risk, and had higher baseline caudate DRD2 availability. The elevated caudate DRD2 among dropouts has been attributed to disproportionate attrition (43%) from a previously identified subgroup characterized by low cognition but high DRD2 levels (Karalija et al., 2022). The study was approved by the Swedish Ethical Review Authority (Umeå, Sweden) and conducted in accordance with the Declaration of Helsinki. Written informed consent was obtained from all participants before any testing.

During the first wave of data collection, participants visited the laboratory on 2 non-consecutive days (the typical interval between visits was 2 days). On the first day, participants completed one part of the cognitive testing and underwent structural and functional MRI scanning. Between the two visits, the participants filled out a questionnaire on sociodemographic, personality, and lifestyle factors (e.g., leisure activity engagement). On the second visit, participants completed the cognitive testing, partook in medical anamnesis and testing of physical parameters, and underwent a PET scan. The procedure for the second wave of data collection was identical to the procedure for the first wave. Time between the first and second data collection waves was 5 years for most of the sample (*M* = 60.1 ± 0.6 months for PET and 60.0 ± 0.4 months for MRI).

### 2.2 Cognitive assessment

Cognitive abilities (working memory, episodic memory, and perceptual speed) were each measured with three tasks. Working memory was assessed using letter updating, numerical 3-back, and spatial updating; episodic memory was assessed using word recall, number–word recall, and object–position recall; and perceptual speed was assessed using letter comparison, number comparison, and figure comparison. For detailed descriptions of the cognitive tasks and their implementation, see Nevalainen et al. (2015). For each task, sum scores were computed across the total number of blocks or trials.

### 2.3 Structural MRI

MRI was performed with a 3 tesla Discovery MR 750 scanner (General Electric, Milwaukee, WI) at both time points. T1-weighted images were obtained with echo time 3.2 milliseconds, flip angle 12°, repetition time 8.19 milliseconds, 176 slices with thickness 1.0 mm, and field of view 25.0 cm with resolution 0.98 mm upsampled to 0.49 mm. The longitudinal image processing pipeline in FreeSurfer, version 6.0, was used to process T1-weighted images and derive estimates of gray matter, white matter, and lateral ventricle size. Subcortical gray matter segmentations were used to define regions of interest (ROIs) for DRD2 assessment. Our primary ROIs were the caudate and putamen, which are well characterized in terms of D2-like receptor availability and have been the primary targets in most prior PET studies of dopamine and aging (Karalija et al., 2022; Karrer et al., 2017).

### 2.4 PET imaging

A 55-minute, 18-frame dynamic PET scan was acquired during rest after an intravenous bolus injection of approximately 250 MBq [^11^C]raclopride (baseline: 263.5 ± 19.0 MBq; follow-up: 260.2 ± 15.0 MBq). An attenuation CT scan (20 mA, 120 kV, 0.8 seconds/revolution) preceded ligand injection. Attenuation and decay-corrected images (47 slices, field of view = 25 cm, 256 × 256-pixel transaxial images, voxel size = 0.977 × 0.977 × 3.27 mm^3^) were reconstructed with the iterative algorithm VUE Point HD-SharpIR (GE; 6 iterations, 24 subsets, 3.0 mm postfiltering; full width at half maximum: 3.2 mm). PET images were motion corrected and co-registered with the structural T1-weighted images from the corresponding session (baseline and follow-up) using the Statistical Parametric Mapping software (SPM12). As a source for co-registration, the sum of the first five frames was used. PET images from both time points were co-registered with the baseline T1 image for three participants (no MRI at follow-up). Two individuals declined to undergo PET at follow-up. Because age-related gray matter loss and ventricular expansion can bias PET estimates of receptor availability (Greve et al., 2016; Smith et al., 2019), PVE correction was applied to minimize contamination from surrounding tissue. Regional PVE correction was conducted using the symmetric geometric transfer matrix implemented in FreeSurfer (Greve et al., 2014, 2016). An incremental PVE-correction approach was used in which (1) the initial correction was achieved using resolution modeling in the iterative image reconstruction procedure (SHARP-IR) and (2) the remnant PVE was controlled for using the ROI-based geometric transfer matrix approach. The size of the secondary correction kernel was estimated empirically (point spread function of 2.5 mm; isotropic) to achieve a similar level of correction as earlier (Smith et al., 2019). FreeSurfer segmentations and preprocessed PET data were used to estimate PVE-corrected regional radioactivity concentrations per ROI and time frame. PVE-corrected non-displaceable binding potential BP_ND_ estimates were calculated with the multilinear reference tissue model (MRTM) on dynamic PVE-corrected data, with cerebellar gray matter as reference region.

### 2.5 Education and leisure activities

A self-report questionnaire on sociodemographic, personality, and lifestyle factors was developed for the purpose of the COBRA study and tailored to life in northern Sweden. For the lifestyle part of the questionnaire, participants answered questions concerning their engagement in physical, cognitive, and social leisure activities. For each leisure activity subdomain, a list of activities was presented (e.g., jogging), and participants were asked to indicate how many hours (1–14 hours with 1-hour increments, or 15+ hours) they would engage in each activity during a typical summer week. Summer was chosen as a reference season because opportunities for physical activity engagement vary substantially across seasons in northern Sweden, and a fixed reference season thus ensures that longitudinal comparisons reflect behavioral changes rather than seasonal variation. In total, 15 physical activities (e.g., walking, dancing, strength training, garden work), 18 cognitive activities (e.g., reading fiction, playing board games, solving crosswords, driving a car), and 10 social activities (e.g., meeting friends, spending time with family members, participating in organized community groups) were included in the questionnaire. For each activity subdomain, a frequency sum score was computed as the total reported hours per week across all subdomain activities. For the physical and cognitive activities, participants were also asked to rate, on a Likert scale ranging from 1 (“not at all demanding”) to 5 (“extremely demanding”), how demanding they perceive each activity to be. These items primarily target subjective effort. Following Köhncke et al. (2018), who carried out similar analyses for the baseline sample and used the term *intensity* for this measure, we adopt the same label here. We included only ratings from individuals who performed a given activity at least 1 hour/week, surmising that activities performed regularly would yield more reliable judgments concerning their perceived intensity compared with activities performed only rarely. For the full questionnaire, see the Supplementary Materials.

### 2.6 Data preparation

Data for three individuals were excluded due to imperfect segmentation of magnetic resonance images and PET/MR image co-registration (*n* = 2) or deviant brain structure (*n* = 1). For the [^11^C]raclopride PET measures, univariate outliers were defined as > 3 SD below the mean and excluded as listwise deletions per ROI (in total, *n* = 5 cases were deleted). For the cognitive measures, two participants had missing data for individual cognitive tasks at T1, and nine participants had missing data on diverse cognitive tasks at T2, with the missingness caused by technical or handling failure during data collection. Univariate outliers for the cognitive data were identified using the outlier labeling rule (Hoaglin & Iglewicz, 1987; Tukey, 1977) with a G-factor of 3. Two participants exhibited scores exceeding the theoretical maximum for specific cognitive tasks, likely due to scoring errors, and the affected values were replaced by the corresponding score from the other block of the task. Multivariate outliers, comparing scores across blocks within the same task, were identified according to the Mahalanobis’ distance (Mahalanobis, 1936). Seven participants exhibited outlier scores on individual blocks. These scores were replaced by scores from other blocks, or, where applicable, the mean of the other blocks. For the leisure activity measures, extreme values on the physical activity intensity variable were winsorized at ±3.29 SD, affecting one participant at T1. After data preparation, the effective sample size was *n* = 176 at T1 and *n* = 126 at T2.

### 2.7 Statistical analyses

Structural Equation Modeling (SEM) with Bayesian estimation was used for the main analyses. An advantage of SEM is that between-individual variance in a construct (e.g., working memory) can be modeled by extracting the common variance across multiple indicators (e.g., three tasks measuring working memory) to form a latent factor. This approach partials out indicator-specific variance and measurement error, thereby mitigating issues with measurement unreliability (Kline, 2011; Lei & Wu, 2007; MacCallum & Austin, 2000). Bayesian SEM, in turn, incorporates information about the model parameters into the model by assigning priors (researcher-specified probability distributions) to each parameter to represent their plausible values. During model estimation, priors are updated in light of the data, thus yielding posterior probability distributions for each parameter and forming the basis for the main results. Bayesian SEM can provide more detailed information about model parameters and improved performance in small samples, and offers more relaxed distributional requirements and fewer restrictions on model specification and model complexity compared with frequentist SEM (Depaoli, 2021; McNeish, 2016; Muthén & Asparouhov, 2012). Table 1 displays the priors used for our analyses.

**Table 1.** Prior parameters.

		Prior parameters	
Parameter type	Prior distribution	Main analyses	Sensitivity analyses
Factor loadings	Normal	*M* = 0.7, *SD* = 1	*M* = 0.7, *SD* = 5
Factor intercepts	Normal	*M* = 0, *SD* = 2	*M* = 0, *SD* = 5
Factor variances	Gamma	Shape = 1, rate = 1	Shape = 1, rate = 0.25
Manifest intercepts	Normal	*M* = 0, *SD* = 2	*M* = 0, *SD* = 5
Manifest variances	Gamma	Shape = 1, rate = 1	Shape = 1, rate = 0.25
Correlations	Beta	α = 2, β = 2	α = 1, β = 1

*Note.* The Normal distribution is parameterized by mean (*M*) and standard deviation (*SD*); the Gamma distribution by shape and rate; and the Beta distribution by α and β. Sensitivity analyses used more diffuse priors to assess robustness.

**Fig. 1.**
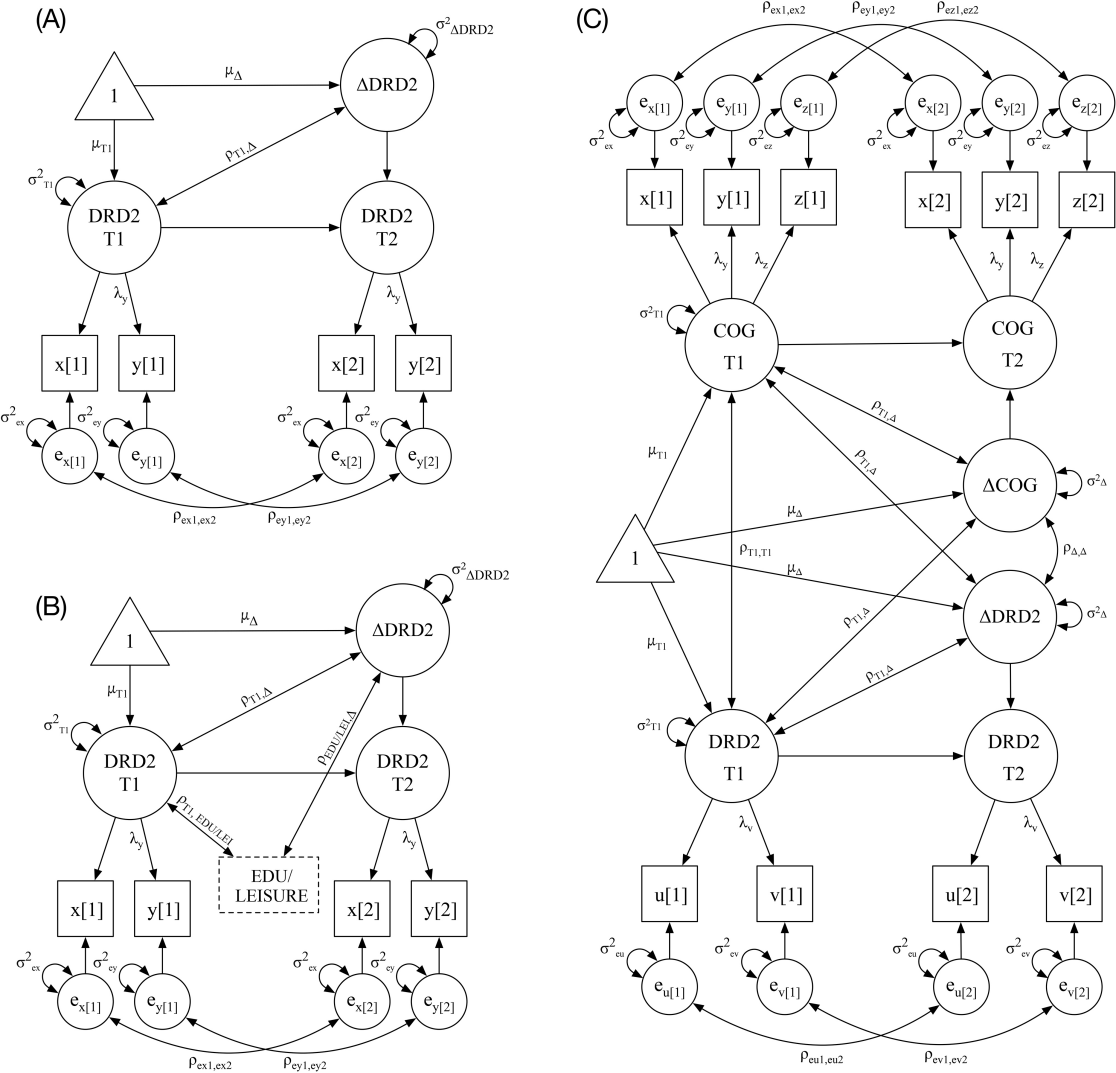
Graphical representations of the latent change score models used in the present study. (A) Univariate DRD2 latent change score model. (B) DRD2 latent change score model with an observed exogenous variable (education or a leisure activity variable; depicted as a dashed rectangle), where the parameters of primary interest are the covariances between the exogenous variable and DRD2 baseline ($\rho_{T1,\text{EDU/LEI}}$ ) and DRD2 change ($\rho_{\text{EDU/LEI},\Delta }$ ). (C) Bivariate DRD2–cognition latent change score model, in which the change–change correlation (ρ_Δ, Δ_) is the parameter of primary interest. All unlabeled parameters are fixed to 1. Manifest (i.e., observed) variables are represented by squares, latent variables by circles, regression weights by one-headed arrows, variances and covariances by two-headed arrows, and the triangle represents the constant used to model means and intercepts.

Longitudinal measurement invariance was examined for each cognitive ability using Bayesian Confirmatory Factor Analysis (CFA; Putnick & Bornstein, 2016; Van de Schoot et al., 2012). Scalar invariance was obtained for all three cognitive abilities, indicating that any observed changes in cognitive performance can be attributed to changes in the cognitive abilities rather than to changes in the psychometric properties of the tasks used to measure them (Supplementary Table S1). For our main analyses, we estimated a set of univariate and bivariate Latent Change Score Models (LCSMs; Ghisletta & McArdle, 2012; Kievit et al., 2018; Matusik et al., 2021; McArdle, 2001, 2009). Figure 1 displays graphical representations of the model templates as they were implemented in the current study. Separate univariate LCSMs were estimated for each DRD2 ROI (Fig. 1A). The models assumed a latent factor at baseline (T1), representing baseline levels of a DRD2 ROI, and an analogous latent factor at follow-up (T2). For the DRD2 ROIs, the latent factors were formed from the left and right hemispheres of each ROI (Kievit et al., 2018; Raz et al., 2005). Education and leisure activity variables (physical activity frequency, physical activity intensity, cognitive activity frequency, cognitive activity intensity, and social activity frequency) entered into the models as exogenous variables, with freely estimated correlations with T1 factors (baseline levels) and latent change factors.

To model the DRD2–cognition change–change correlations, we estimated a bivariate LCSM (Fig. 1C) for each combination of a DRD2 ROI (caudate and putamen) and a cognitive ability (working memory, episodic memory, and perceptual speed). For the cognitive abilities, the sum scores on the cognitive tasks were used as indicators to form latent factors of each ability. Using this approach, the T2 factor is defined as the unit-weighted sum of the corresponding T1 factor and a latent change factor representing the longitudinal change between T1 and T2. Factor loadings and intercepts were constrained to equality between time points in all models, reflecting scalar measurement invariance. Using this approach, the change–change correlations are represented as correlations between the two latent change factors in each bivariate model. Similarly, to examine DRD2–leisure activity change–change correlations, a bivariate LCSM (not depicted in Fig. 1) was estimated for each combination of a leisure activity measure and a DRD2 ROI. Lastly, we conducted a set of moderation analyses in which we examined whether the DRD2–cognition change–change correlations are moderated by education. This involved re-estimating the bivariate LCSMs as multigroup models, where the participants were split into a lower and higher education group using a median split. For completeness, the same approach was used to examine moderating influences by any of the leisure activity measures. Aside from the multigroup specification, the models estimated for the moderation analyses were identical to the initial bivariate LCS models.

All variables included in the models were standardized (transformed to z scores) before model estimation to facilitate prior specification. The follow-up (T2) variables were standardized using the baseline (T1) means and standard deviations according to the formula $\left(x_{i,T2}-{\bar{x}}_{T1}\right)/SD_{T1}$ , thus capturing longitudinal change. Priors were designed to be weakly informative, reflecting a moderate level of uncertainty around the parameters. To assess the robustness of our results to alternative prior specifications, a second set of more diffuse priors was specified and used for sensitivity analyses (Table 1).

Missing data were handled using full information Bayesian estimation (Schafer & Graham, 2002), in which missing values are treated as additional parameters and sampled within each MCMC iteration (Bayesian data augmentation). This approach uses all available information in the data and allows participants with partial missing data to be included without listwise exclusion, while reducing bias from longitudinal selectivity under the assumption that data are missing at random. Full information estimation generates less biased population estimates than other widespread procedures for dealing with missing values (e.g., listwise deletion, regression imputation, or mean imputation; Schafer & Graham, 2002).

All analyses were performed using R (v4.5.3; R Core Team, 2026). Descriptive statistics for the observed variables are summarized in Table 2. Bayesian SEM models were estimated using the blavaan R package with Stan (Merkle & Rosseel, 2018; Merkle et al., 2021), utilizing Stan’s No-U-Turn Sampler (Hoffman & Gelman, 2014) with random starting values for all model parameters. All our model estimations used 3 Markov chains with 2,000 burn-in iterations and 8,000 post-burn-in iterations each. Convergence was monitored using the $\hat{R}$ statistic (Brooks & Gelman, 1998; Gelman & Rubin, 1992) with a conservative cutoff set at 1.01, as well as by inspecting all trace plots and autocorrelation plots (Depaoli, 2021). All 270 estimated models converged: $\hat{R}$ values ranged from 1.000 to 1.004, effective sample sizes (ESS) ranged from 2,070 to 9,466. Data-model fits, evaluated using the posterior predictive *p*-values, Bayesian $\hat{\Gamma }$ , and Bayesian *M_c_*, indicated good-to-excellent fit. Additional details on modeling strategy, measurement invariance testing, model fit statistics, and prior specification are available in the Supplementary Materials.

**Table 2.** Descriptive statistics for the sample characteristics, leisure activity measures, cognitive tasks, and DRD2 availability measures.

	Baseline (*n* = 176)				Follow-up (*n* = 126)			
Variable	Mean	*SD*	Skew	Kurtosis	Mean	*SD*	Skew	Kurtosis
Age (years)	66.20	1.22	-0.17	-1.06	71.17	1.22	-0.14	-1.05
Education (years)	13.29	3.52	0.45	0.15	13.37	3.47	0.28	0.02
Sex (% female)	45%	–	–	–	47%	–	–	–
BMI (kg/m^2^)	26.16	3.56	0.81	1.26	–	–	–	–
Physical activity (hours/week)	22.31	12.06	1.18	1.67	21.80	10.82	1.00	0.71
Physical activity intensity	1.67	0.65	0.88	0.30	1.72	0.65	0.51	-0.70
Cognitive activity (hours/week)	34.83	16.16	1.10	2.20	36.15	17.66	1.01	0.98
Cognitive activity intensity	1.43	0.42	1.34	1.74	1.42	0.46	1.46	2.01
Social activity (hours/week)	32.48	16.21	1.15	1.64	30.54	14.70	0.79	0.23
WM: Letter updating	33.26	8.26	-1.08	0.98	33.18	8.32	-0.82	0.10
WM: Numerical 3-back	77.95	16.57	-0.40	0.01	77.98	16.26	-0.21	-1.08
WM: Spatial updating	13.09	6.13	-0.07	-0.26	12.48	6.64	0.07	-0.66
EM: Word recall	12.98	4.16	0.33	0.40	12.75	4.23	0.54	0.45
EM: Number–word recall	3.60	2.45	0.94	0.87	3.77	2.39	0.68	0.35
EM: Object–position recall	12.31	3.62	-0.04	-0.34	12.31	3.95	0.14	-0.67
PS: Letter comparison	63.52	14.92	0.57	-0.04	63.27	14.64	0.35	-0.42
PS: Number comparison	71.03	14.29	0.54	0.14	70.54	14.10	0.40	0.25
PS: Figure comparison	29.59	5.44	0.54	-0.17	29.56	6.04	0.91	0.90
Caudate DRD2 (left)	2.70	0.46	-0.68	1.17	2.58	0.51	-0.72	1.09
Caudate DRD2 (right)	2.80	0.44	-0.64	2.96	2.63	0.51	-1.00	2.53
Putamen DRD2 (left)	3.75	0.38	0.00	-0.45	3.65	0.41	-0.09	-0.39
Putamen DRD2 (right)	3.79	0.39	-0.38	0.24	3.68	0.42	-0.37	0.35

*Note.* WM = Working Memory; EM = Episodic Memory; PS = Perceptual Speed; DRD2 = Dopamine D2-like receptor availability (BP_ND_).

In interpreting our Bayesian results, we rely on two complementary criteria. First, we use 95% Highest Density Intervals (HDIs), where exclusion of zero in the HDI constitutes credible evidence for the presence of a non-zero effect. Second, we compute the posterior probability of a directional effect (pD), which quantifies the proportion of the posterior distribution above or below zero. These two criteria answer different questions about the same posterior distribution: The HDI indicates whether a null effect remains credible, while the pD indicates the probability that the effect is in a given direction. As such, a high pD value (e.g., pD_>0_ = .95) indicates that the direction of the association is highly probable even if the 95% HDI spans zero. We report both metrics throughout the Results section to provide a comprehensive characterization of the evidence.

## 3 Results

### 3.1 Longitudinal 5-year changes in DRD2 availability and cognitive performance

Table 3 presents the posterior estimates for the 5-year longitudinal changes in DRD2 availability and cognition. In line with previous reports from the COBRA data (Karalija et al., 2022), the posterior means for the group-level changes in DRD2 availability were negative across both striatal regions, with 95% HDIs excluding zero, thus providing strong evidence for 5-year DRD2 declines at the group level. The declines in the two striatal regions were approximately equal. On the cognitive side, the group-level posterior means revealed small declines across all three cognitive abilities; however, all three 95% HDIs narrowly extended across zero. The posterior probabilities of decline (pD_<0_) were .97 for WM, .88 for EM, and .85 for PS, suggesting that negative change is highly probable for WM and moderately probable for EM and PS, but that negligible or very small positive changes cannot be ruled out.

**Table 3.** Standardized latent change score estimates for the regional DRD2 availability and cognitive abilities.

	Group-level change		Individual differences in change	
Latent change (Z)	Posterior mean	95% HDI	Posterior mean	95% HDI
Caudate DRD2	-0.18	[-0.30, -0.06]	0.16	[0.00, 0.32]
Putamen DRD2	-0.20	[-0.30, -0.09]	0.22	[0.12, 0.32]
Working memory	-0.12	[-0.25, 0.00]	0.19	[0.01, 0.38]
Episodic memory	-0.06	[-0.17, 0.04]	0.05	[0.00, 0.14]
Perceptual speed	-0.05	[-0.14, 0.04]	0.19	[0.12, 0.27]

*Note.* All estimates represent Bayesian posteriors. *Group-level change* refers to posterior estimates of the average change across individuals, while individual differences in change reflect posterior estimates of the change score standard deviation (SD), capturing individual differences in within-person change. *Posterior means* represent the central tendency of the posterior distributions. *95% HDIs* (Highest Density Intervals) represent the range containing the most credible 95% of the posterior distribution. 95% HDIs spanning zero indicate that effects in either direction remain credible.

**Fig. 2.**
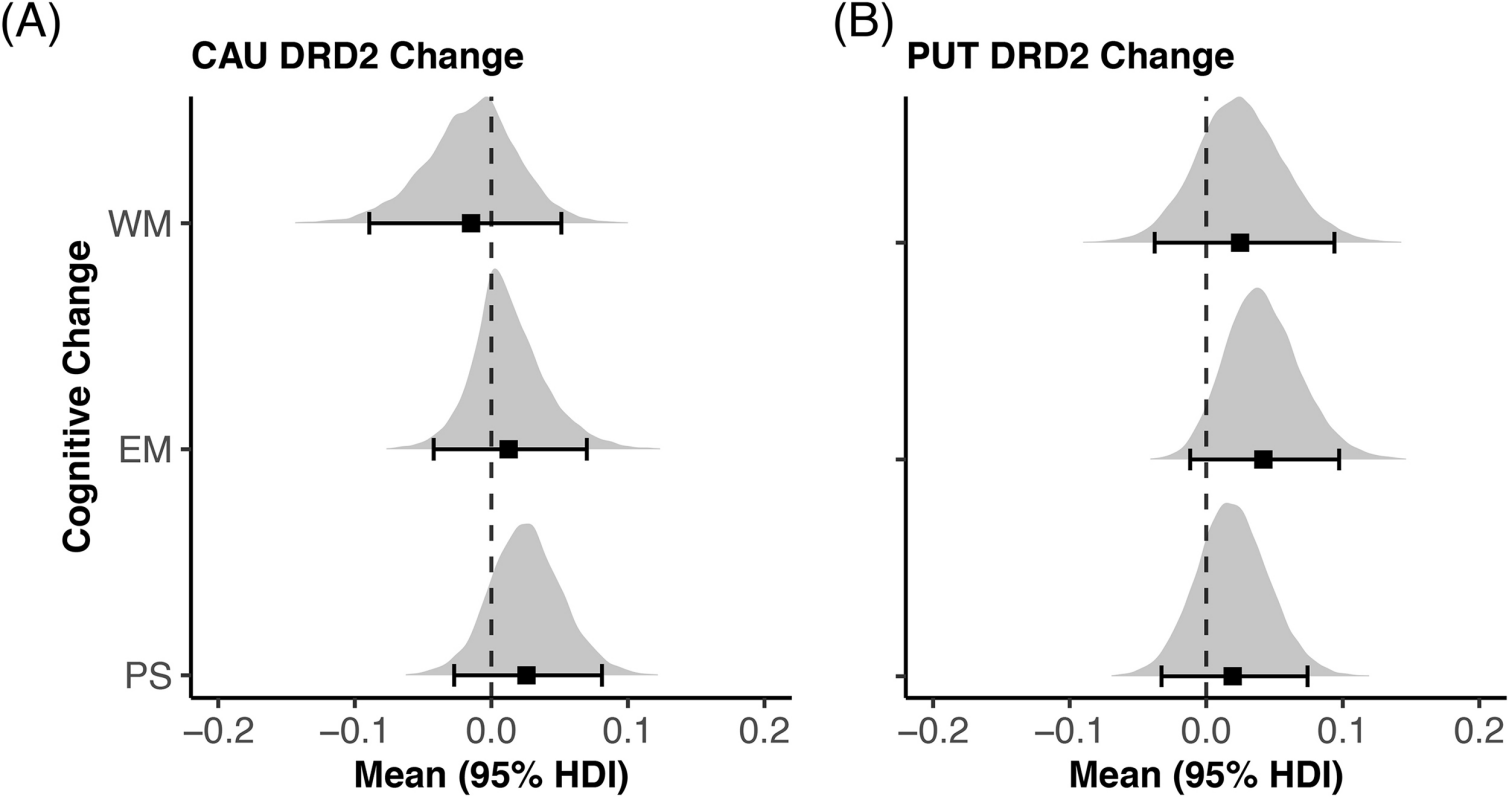
Correlations between changes in striatal DRD2 availability and changes in cognition. Points indicate posterior means, whiskers represent 95% Highest Density Intervals (HDIs), and shaded density plots show the full posterior distributions. Panel A: Caudate DRD2 Change. Panel B: Putamen DRD2 Change. HDIs spanning zero indicate that effects in either direction remain credible. DRD2 = Dopamine D2-like receptor availability; CAU = Caudate; PUT = Putamen; WM = Working Memory; EM = Episodic Memory; PS = Perceptual Speed.

Figure 2 presents the posterior estimates for the DRD2–cognition change–change correlations. The correlations were consistently small across both striatal ROIs and cognitive abilities (all posterior mean |*r*| <.05), with all 95% HDIs spanning zero. A positive change–change correlation was most probable for the putamen–EM pair (pD_>0_ = .95, *M* = .04, 95% HDI = [−.01, .10]) and least supported for the caudate–WM pair (pD_>0_ = .34), for which the posterior distribution slightly favored a negative association (*M* = −.01, 95% HDI = [−.09, .05]). Except for the caudate–WM pair, all *r’*s were positive based on posterior means.

### 3.2 Associations between DRD2 availability, education, and leisure activities

**Fig. 3.**
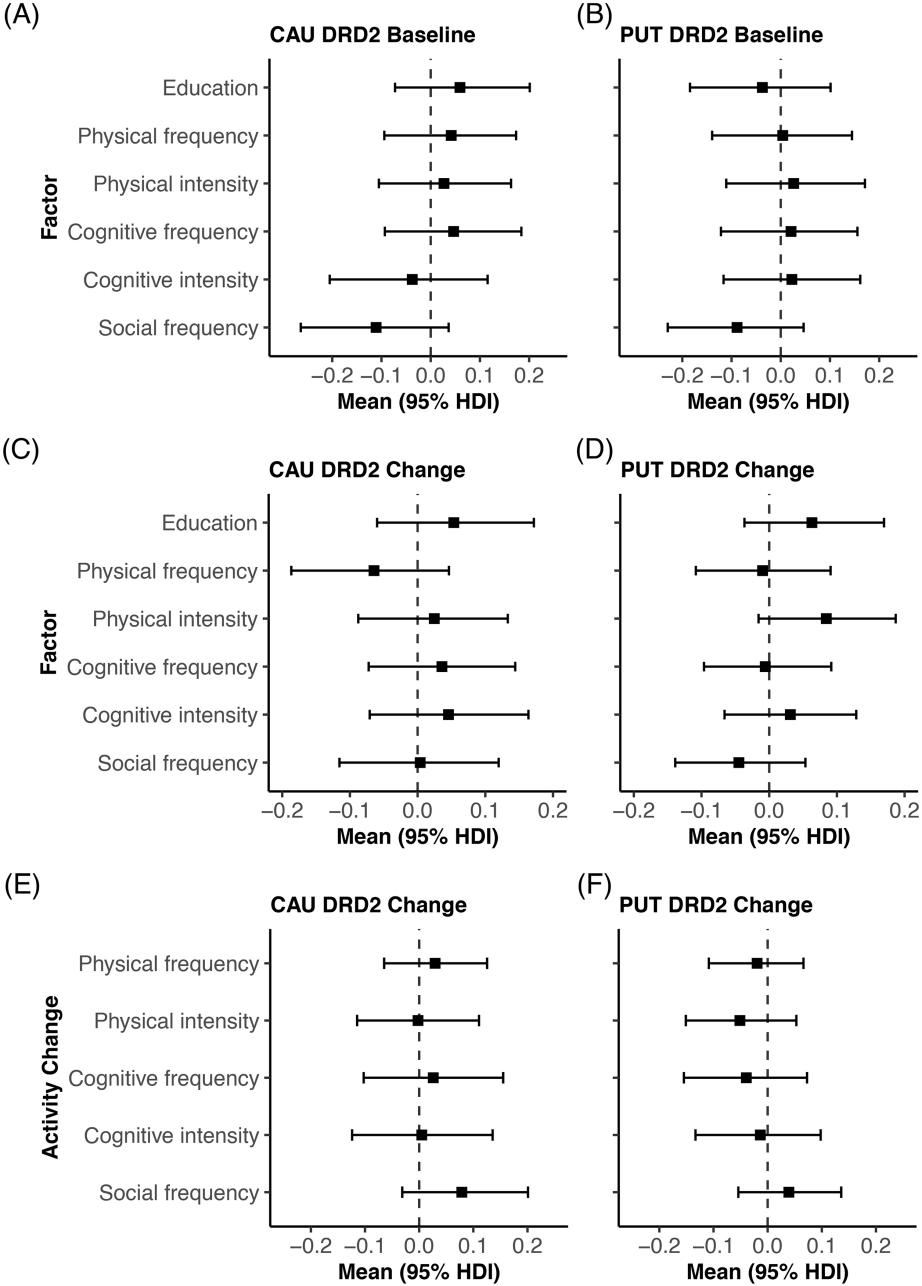
Associations between education, leisure activities, and striatal DRD2 availability. Panels (A, B) show correlations with baseline DRD2 levels; panels (C, D) show correlations with longitudinal DRD2 changes; panels (E, F) show change–change correlations between DRD2 changes and leisure activity changes. Points indicate posterior means, whiskers represent 95% HDIs.

Figure 3 shows the estimated correlations between education, leisure activities, and both baseline and change estimates of DRD2 availability in the caudate and putamen. As shown in the figure, all correlations were small (all posterior mean |r| <.12), and all 95% HDIs spanned zero. For the baseline DRD2 levels (Fig. 3A, B), negative correlations between social activity frequency and baseline DRD2 availability were the most notable, with pD_<0_ values of .93 (caudate) and .90 (putamen) (*M* = −.11, 95% HDI [−.26, .04] for caudate; *M* = −.09, 95% HDI [−.23, .05] for putamen). With regard to the associations between 5-year DRD2 changes and baseline measures of education and leisure activities shown in Figure 3C and D, we emphasize that a positive correlation between a factor (e.g., education) and changes in DRD2 entails that an increase in the factor is associated with less DRD2 decline—similarly, a negative correlation entails that an increase in the factor is associated with more DRD2 decline. Small positive correlations between DRD2 changes and education were moderately probable across both striatal ROIs (*M* = .05, 95% HDI [−.06, .17], pD_>0_ = .83 for caudate; *M* = .06, 95% HDI [−.04, .17], pD_>0_ = .89 for putamen), suggesting that higher education may be weakly associated with less DRD2 decline. Physical activity frequency showed a moderately probable small negative association with caudate DRD2 change (*M* = −.06, 95% HDI [−.19, .05], pD_<0_ = .87), indicating that higher physical activity frequency may be associated with greater caudate DRD2 decline, although the unexpected direction warrants cautious interpretation. Notably, physical activity intensity showed a positive association with putamen DRD2 change (*M* = .08, 95% HDI [−.02, .19], pD_>0_ = .95), suggesting that higher self-rated physical activity intensity is probably associated with less putamen DRD2 decline, although the association is likely small. Figure 3E and F shows the change–change correlations between the 5-year changes in DRD2 availability and the corresponding 5-year changes in leisure activities. A positive correlation would indicate that an increase in a leisure activity measure is associated with less regional DRD2 decline. All estimated correlations were small (all posterior mean |r| ≤.08), and 95% HDIs spanned zero. The strongest directional probability among the activity change–change associations was for social activity frequency and caudate DRD2 change (*M* = .08, 95% HDI = [−.03, .20], pD_>0_ = .92).

In sum, associations between education, leisure activities, and both baseline levels and 5-year changes in DRD2 availability were small and uncertain in terms of magnitude, and all 95% HDIs spanned zero. The most credible directional associations were a positive correlation between physical activity intensity and putamen DRD2 change (pD_>0_ = .95), negative correlations between social activity frequency and baseline striatal DRD2 (pDs_<0_ = .90–.93), a positive caudate DRD2–social activity change–change correlation (pD_>0_ = .92), and positive correlations between education and DRD2 changes (pDs_>0_ = .83–.89). If education or leisure activities are associated with baseline levels or 5-year changes in striatal DRD2 availability, the effects are likely negligible or small.

### 3.3 Moderation analyses

**Fig. 4.**
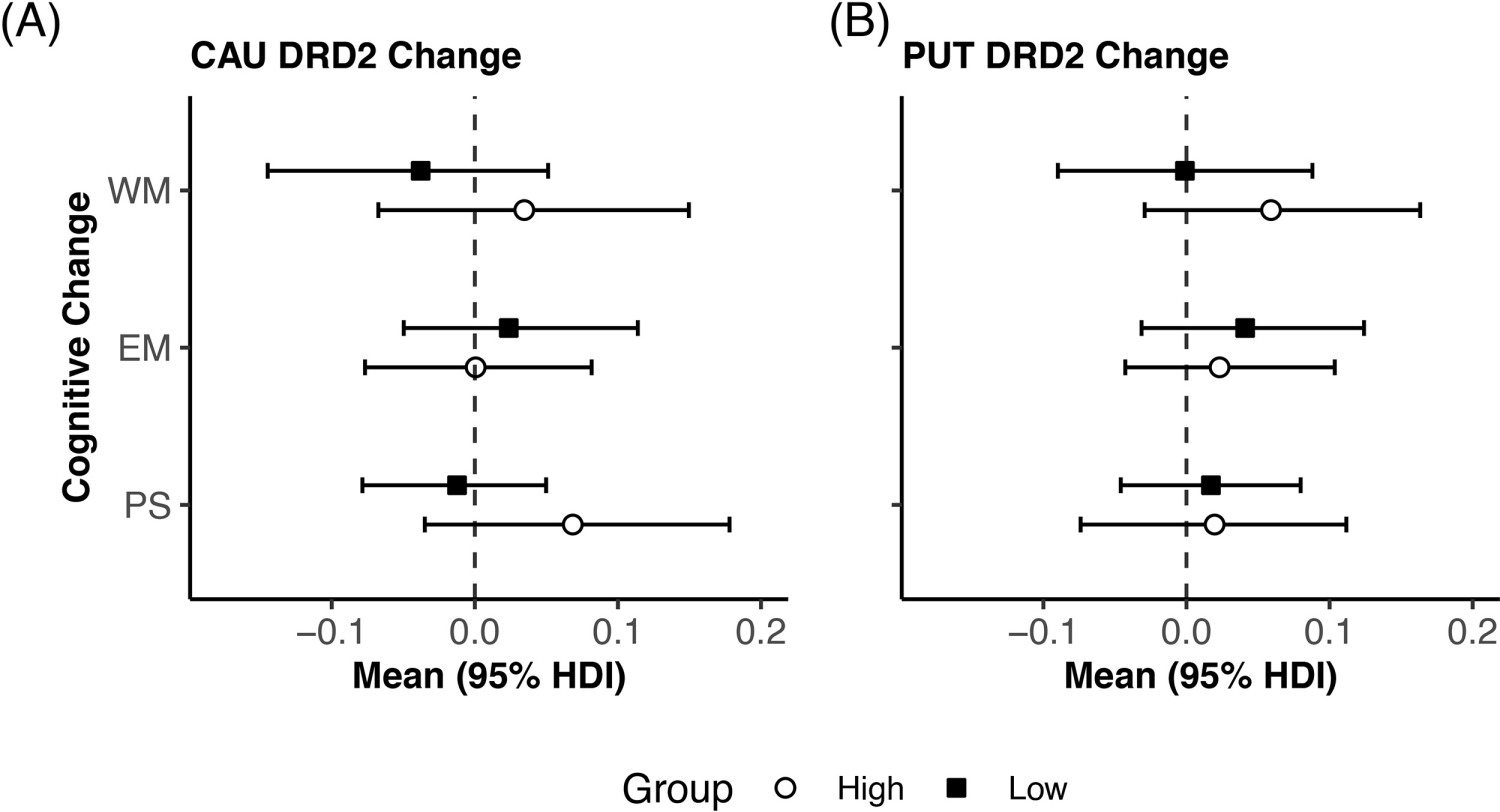
Correlations between changes in striatal DRD2 availability and changes in cognition, estimated separately for the low and high education groups (median split). Panel A: Caudate DRD2 Change. Panel B: Putamen DRD2 Change. Points indicate posterior means, whiskers represent 95% HDIs. Filled squares = low education; open circles = high education.

Figure 4 presents the results of the moderation analysis for education, showing the estimated DRD2–cognition change–change correlations separately for the low education and high education groups of the median split. Moderation is expressed as between-group differences in correlation estimates. As shown in Figure 4, the between-group differences in posterior mean *r’*s are small across all models (|*r*_diff_| ≤.08), with considerable overlap between the 95% HDIs for each group. The direction of the moderation is inconsistent, with some pairs showing stronger correlations in the high education group (caudate–EM, putamen–EM), and the remaining pairs showing stronger correlations in the low education group. As such, the results reveal no systematic pattern for a cognitive reserve effect, which would predict uniformly larger correlations in the low education group: If a moderating effect of education exists, the effect is likely negligible or small. For completeness, we also examined moderation effects of the leisure activity measures. No strong evidence for moderation was obtained. Full results are reported in the Supplementary Materials.

### 3.4 Supplementary analyses

Analyses of cognitive abilities, examining associations between education, leisure activities, and both baseline levels and changes in cognition, were conducted. Because our emphasis in this study lies on DRD2, we report results briefly here, with full details in the Supplementary Materials (Supplementary Figs. S1 and S2). There was strong evidence for positive correlations between education and baseline levels of working memory performance (*M* = .20, 95% HDI [.06, .35]), episodic memory performance (*M* = .26, 95% HDI [.13, .39]), and perceptual speed (*M* = .16, 95% HDI [.03, .30]), as well as for positive correlations between physical activity intensity and baseline levels of both working memory performance (*M* = .22, 95% HDI [.07, .37]) and episodic memory performance (*M* = .18, 95% HDI [.05, .31]). Cognitive activity frequency was also positively associated with baseline episodic memory (*M* = .17, 95% HDI [.04, .31]). No other association was strongly supported.

Our main analyses compute mean physical activity intensity across all 15 listed activities. In a previous cross-sectional analysis on the COBRA baseline data, Köhncke et al. (2018) used a subset of the five most commonly performed activities (walking, cycling, jogging, strength training, sports), finding a significant correlation between a latent factor of physical intensity and caudate DRD2 availability (*r* = .32, *p* < .001 in their frequentist analysis). To examine whether the inconsistency between the weak cross-sectional association in our main analysis and the Köhncke et al. (2018) result reflects this difference in construct composition, we estimated the physical intensity models using the mean intensity across the same subset of five activities. This analysis recovered positive associations with baseline DRD2 availability in the caudate (pD_>0_ = .97) and putamen (pD_>0_ = .95). The five-item physical intensity analyses yielded no strong evidence for influences of baseline physical intensity on DRD2 change (all posterior mean |*r*| ≤.04, all pD_>0_ ≤ .77). A moderately probable but small caudate DRD2–physical intensity change–change correlation was obtained (*M* = .08, 95% HDI [−.07, .25], pD_>0_ = .85); we note, however, that this model showed a borderline posterior predictive *p*-value (PPP = .087), slightly below the conventional .10 threshold (see Supplementary Materials, Section S4), and the corresponding estimate should, therefore, be interpreted with additional caution. Full results are reported in Supplementary Table S2.

Sensitivity analyses using an alternative set of priors (Table 1) showed minimal changes in posterior means and 95% HDIs for the target parameters across all models (Supplementary Figs. S3–S6). No substantive conclusions were affected, indicating the robustness of our findings to prior specification. Additionally, supplementary analyses controlling for sex and BMI yielded results very similar to the main analyses (all posterior means differed by less than .02 from the unadjusted estimates). The Supplementary Materials contain detailed results for the sensitivity and covariate analyses.

## 4 Discussion

The identification of protective and risk factors for neurocognitive aging represents an area of strong research interest. The current study examined the associations of education and leisure activity engagement with baseline levels and 5-year changes in DRD2 availability in healthy older adults, along with their potential moderating influences on DRD2–cognition change–change correlations. Overall, our findings suggest that such associations are likely negligible or small. We first briefly discuss the univariate and bivariate changes in DRD2 and cognition, and then proceed to discuss the main findings concerning education and leisure activities.

Consistent with previous studies examining age-related changes in striatal DRD2 availability (Antonini et al., 1993; Karrer et al., 2017), including previous analyses conducted within the COBRA project (Karalija et al., 2022; Lundgren et al., 2025), our results provide strong evidence for age-related declines in striatal DRD2 availability in healthy older adults: Average standardized 5-year change was -0.18 for caudate and -0.20 for putamen based on latent posterior means, with marked individual differences in change. The modest cognitive changes, with average standardized 5-year changes ranging between −0.05 and −0.12 across the three abilities, are consistent with the relatively brief 5-year interval, over which age-related cognitive decline in healthy adults is typically small (Rönnlund et al., 2005). Notably, substantial individual differences in the magnitude and direction of cognitive change were observed across all three abilities, providing meaningful variance for examining the DRD2–cognition change–change correlations. The directional probabilities indicated that positive change–change correlations were probable for several DRD2–cognition pairs (e.g., putamen–EM: pD_>0_ = .95), but even the most probable associations were small (*r’*s ≤ .05 based on the posterior means). Over short intervals such as 5 years, changes in DRD2 availability and cognitive decline appear only weakly coupled in the current age group. This is consistent with the broader brain–cognition change literature, which has tended to reveal small or uncertain associations (Nyberg et al., 2012; Oschwald et al., 2019; Salthouse, 2011), although DRD2–cognition change correlations become detectable over longer intervals (Lundgren et al., 2025) and may be stronger in certain subgroups depending on cerebrovascular health (Lövdén et al., 2018), genetic variation (Karalija et al., 2019), and propagation of D2 decline across DA pathways (Karalija et al., 2024; Papenberg et al., 2025).

DRD2 associations with education were small and uncertain, and we found no credible evidence for an association between education and baseline levels of DRD2 availability. While the directional probabilities moderately favored positive correlations between education and DRD2 change in both striatal regions (pDs_>0_ = .83–.89), tentatively suggesting that higher education may be associated with less DRD2 decline, the correlations were small (*r’*s = .05–.06 based on posterior means) and the 95% HDIs included zero. Studies in recent years have challenged the view that higher education slows brain aging, with evidence suggesting minimal effects on rates of brain atrophy (e.g., Fjell et al., 2025; Lövdén et al., 2023; Nyberg et al., 2021). Our results extend these findings to the domain of neurotransmitter systems: To the extent that education mitigates age-related losses of DRD2 availability, the effect is likely very small (*r*^2^ <.004 in our sample). Similarly, we found no credible evidence that education moderates 5-year DRD2–cognition change–change correlations. Relatedly, cognitive reserve theory predicts that higher education buffers cognitive function against adverse brain changes caused by aging, pathology, or injury (Cabeza et al., 2018; Stern et al., 2020). Our results are inconsistent with this prediction, aligning instead with recent studies that found no moderating influence of education on brain–cognition change associations (Fjell et al., 2025; Lövdén et al., 2023). While our findings do not fully rule out a moderating role of education on DRD2–cognition change–change associations, any such effect is likely negligible given the current sample and time interval.

For the physical activity measures, associations were similarly small and uncertain, with all 95% HDIs spanning zero. We found a probable positive association between baseline self-rated physical activity intensity and 5-year DRD2 change in the putamen (*r* = .08 based on posterior mean, pD_>0_ = .95), providing tentative support for the hypothesis that physical activity may mitigate DRD2 loss. Physical exercise is thought to contribute to brain maintenance (Boraxbekk et al., 2016; Dunås et al., 2021; Stillman et al., 2020), but intervention studies examining effects of exercise on changes in DRD2 availability in humans have provided mixed results, possibly in part due to differences in exercise parameters, sample characteristics, control conditions, and differences in [^11^C]raclopride versus [^18^F]fallypride tracer affinity (Jonasson et al., 2019; Robertson et al., 2015; von Cederwald et al., 2023; Wang et al., 2000). Notably, our physical activity intensity construct captures self-rated subjective effort, not objective intensity measures (e.g., accelerometry or heart rate monitoring), and thus reflects individual tendencies to exert greater relative effort during physical activity. This approach differs from compendium-derived metabolic-equivalent weighted intensity measures (e.g., Ainsworth et al., 2011), which assign fixed intensity values based on activity type rather than individual effort within activities.

In contrast to Köhncke et al. (2018), who reported positive associations between self-rated physical activity intensity and baseline striatal DRD2 availability in the COBRA baseline sample using a subset of five commonly performed physical activities, our analyses—which include all 15 physical activities listed in the questionnaire—found no credible association between physical activity intensity and baseline DRD2 in any ROI. The discrepant results likely reflect variation driven by activity selection (i.e., construct composition) rather than by effort levels during shared activities, likely introducing noise that attenuates linear associations. Consistent with this interpretation, we carried out a supplementary analysis in which physical intensity was computed from the same five commonly performed activities as in Köhncke et al. (2018). This analysis recovered the positive associations with baseline caudate and putamen DRD2 availability (pD_>0_ = .95–.97; Supplementary Table S2), but the five-item version of the physical intensity construct in turn lowered the correlation with DRD2 change in the putamen to *r* = .04 (95% HDI = [−.06, .14], pD_>0_ = .77), thus suggesting that this correlation is sensitive to construct composition and should be interpreted cautiously. If physical intensity is associated with reduced DRD2 decline, the effect is likely negligible or small. For physical activity frequency, the only notable directional probability was a small moderately probable negative association with caudate DRD2 change (*r* = -.06 based on the posterior mean, pD_<0_ = .87). A negative association between physical activity and DRD2 change is consistent with either accelerated receptor loss or increased endogenous dopamine levels displacing the radioligand (Jonasson et al., 2019; von Cederwald et al., 2023). The direction of this association is opposite to that observed for physical activity intensity, which may reflect the distinct constructs captured by these measures. There was no credible evidence that frequency or intensity of physical activity moderates the change–change correlations.

For cognitive activities, neither frequency nor intensity showed credible associations with baseline DRD2 levels or 5-year DRD2 change. Engagement in cognitively stimulating leisure activities has been proposed to protect against age-related cognitive decline (Bielak & Gow, 2022; Hertzog et al., 2008), though the evidence base remains equivocal, with consistent level–level associations but limited support for effects on longitudinal cognitive change (Bielak et al., 2012; Salthouse, 2006). Studies linking cognitive leisure activities to brain measures have similarly yielded modest results; in the most comprehensive multimodal MRI study to date, Anatürk et al. (2020) found that cognitive activity levels were associated with executive function, but associations with gray matter volume, white matter microstructure, and white matter lesions were weak and did not survive correction for multiple comparisons. Our null findings extend this pattern to neurochemical markers of brain aging, suggesting that if cognitive leisure activity frequency or intensity influences DRD2 availability, the effect is too small to detect in the current sample. Social activity frequency showed probable negative correlations with baseline striatal DRD2 availability (pDs_<0_ = .90–.93), though the 95% HDIs spanned zero. If genuine, this association could reflect higher tonic dopamine levels in more socially active individuals (consistent with social interaction engaging the mesolimbic reward system) or could reflect trait-level differences in dopaminergic functioning that predispose individuals toward greater social engagement (e.g., Martinez et al., 2010; Reeves et al., 2007; Wacker & Smillie, 2015). Similarly, a small but probable DRD2–social activity change–change correlation was obtained (*r* = .08 based on the posterior mean, pD_>0_ = .92), tentatively indicating that increases in social activity may covary with less caudate DRD2 decline. However, given that all 95% HDIs for social activity associations spanned zero, these results should be interpreted cautiously, and future work with larger samples or over longer time intervals is needed.

In sum, the overall pattern of results across all education and lifestyle variables is one of consistently small and uncertain associations with striatal DRD2 availability. This extends recent findings suggesting minimal effects of education and leisure activities on brain structural aging (e.g., Anatürk et al., 2020; Fjell et al., 2025; Lövdén et al., 2023; Nyberg et al., 2021) to the domain of dopaminergic neurotransmission. Whether this reflects a genuine absence of meaningful associations, or whether such associations might emerge with larger samples, longer follow-up intervals, more precise lifestyle measurement, or in the presence of moderating factors such as cerebrovascular burden (e.g., Johansson et al., 2023; Karalija et al., 2019, 2022; Rieckmann et al., 2016; von Cederwald et al., 2023), remains an open question for future research.

### 4.1 Limitations and future directions

Several limitations should be acknowledged. First, while our sample size is large for a PET study (Karrer et al., 2017), it remains limited from a statistical standpoint, particularly for SEM-based analyses. Although our Bayesian approach makes optimal use of the available data, a larger sample would provide higher precision in the parameter estimates. Second, the 5-year follow-up interval is comparatively brief from a neurocognitive aging standpoint, and may have limited our ability to detect small protective effects that accumulate over longer periods. Third, our measures of leisure activity engagement are based on self-reported questionnaires, which represent imperfect proxies for actual behavior and may be subject to recall inaccuracies, social desirability effects, or inconsistencies in interpretation of the questions. The set of questions used in the questionnaires might not exhaustively cover the physical, cognitive, and social leisure activities that the participants engage in, and results may be sensitive to construct composition. Fourth, the moderation analyses relied on median splits to define education and leisure activity subgroups, which reduces statistical power and precision relative to continuous interaction approaches. Fifth, the analyses focused on whole-region estimates for the caudate and putamen. Finer-grained examination of functional striatal subregions may reveal distinct DRD2 availability profiles and differential associations with lifestyle factors and cognitive processes. Finally, the observational design precludes causal inference. Future research should examine protective and risk factors for DA over longer time intervals, ideally incorporating objective measures of lifestyle (e.g., accelerometry for physical activity) alongside self-reports in order to reduce measurement error. Additionally, assessing lifestyle at earlier time points (e.g., midlife) would help clarify whether long-term lifestyle patterns influence later-life DA integrity and cognitive health.

### 4.2 Conclusions

To our knowledge, this study provides the first comprehensive longitudinal examination of whether education and leisure activities are associated with striatal DRD2 availability and its rate of decline in aging. Despite the breadth of lifestyle variables examined, associations with DRD2 were consistently small and uncertain, extending recent evidence of minimal lifestyle effects on brain structural aging to the domain of dopaminergic neurotransmission. Clarifying whether stronger associations emerge with more precise lifestyle measurement, longer follow-up, or in the presence of cerebrovascular risk factors represents an important direction for future research.

## Supplementary Material

Supplementary Material

## Data Availability

Swedish data-protection laws prohibit us from providing the data in the public domain, but data can be requested from the authors and subsequently transferred for well-defined analysis projects that are in line with the one covered by the original ethical approval. Complete model outputs and code used for the analyses are provided at the OSF repository: https://osf.io/pwy8f/.

## Supplementary Materials

Supplementary Materials include Section S1 (leisure activity questionnaire, translated to English) and Sections S2–S8 (detailed methods, model diagnostics, extended results, and sensitivity analyses). Supplementary material for this article is available here: https://doi.org/10.1162/IMAG.a.1302#supplementary-data.
