# Supplementary material for: Five-year associations among dopamine D2-like receptor loss, cognitive decline, education, and self-reported leisure activities in healthy older adults
Source: Imaging Neurosci (Camb). 2026 Jul 21;4:IMAG.a.1302. doi: 10.1162/IMAG.a.1302 (PMC13389743; doi:10.1162/IMAG.a.1302)
Supplement: Supplementary Material [file IMAG.a.1302_supp.pdf]

Supplementary Materials for: Five–Year Associations Among Dopamine D2–Like Receptor Loss, Cognitive Decline, Education, and Self–Reported Leisure Activities in Healthy Older Adults

Amos Pagin,[1*] Nina Karalija,[2,3] Micael Andersson,[2,3] Lars Nyberg,[2−4] Lars Bäckman,[5] Katrine Riklund,[2,4] Ulman Lindenberger,[6,7] and Martin Lövdén[1]

[1]Department of Psychology, University of Gothenburg

[2]Umeå Center for Functional Brain Imaging, Umeå University

[3]Department of Medical and Translational Biology, Umeå University

[4]Department of Diagnostics and Intervention, Umeå University

[5]Aging Research Center, Karolinska Institute and Stockholm University

[6]Center for Lifespan Psychology, Max Planck Institute for Human Development

[7]Max Planck Centre for Computational Psychiatry and Ageing Research

*Correspondence: amos.pagin@psy.gu.se

June 26, 2026

Introductory Note

Section S1 includes the full leisure activity questionnaire used in the study (translated to English). Sections S2–S8 include detailed methods, model diagnostics, extended results, and sensitivity analyses. Complete model outputs and analysis code are available at the OSF repository: https://osf.io/pwy8f/.

S1. Questionnaire

Below are the questions included in the sociodemographic and lifestyle self-report questionnaire, translated into English. For the questions relating to the frequencies of activities, participants were asked to indicate how many hours (1–14 hours with 1-hour increments, or 15+ hours) they would engage in each activity. For the questions relating to the perceived demands (or intensities) of each activity, participants were asked to rate the intensities on a Likert scale ranging from 1 ("not at all demanding") to 5 ("extremely demanding").

Cognitive Leisure Activities

In a typical week, how many hours do you devote to...

1. Driving a car?
2. Play some type of computer game?
3. Use a computer for something other than playing games?
4. Cooking?
5. Reading non-fiction?
6. Reading fiction?
7. Reading the newspaper?
8. Reading weekly magazines or journals?
9. Attending lectures at a university or other adult education programs?
10. Studying (e.g., language learning) at home?
11. Playing card games?
12. Playing board games (e.g., chess or backgammon)?
13. Playing a musical instrument?
14. Going to museums or art exhibitions?

15. Solving crosswords?
16. Solving puzzles, riddles or problems (e.g., Sudoku)?
17. Writing letters, e-mail, or other text?
18. Doing mathematics (e.g., for work, or managing household finances)?

How cognitively demanding do you find it to...

1. Drive a car?
2. Play some type of computer game?
3. Use a computer for something other than playing games?
4. Cook?
5. Read non-fiction?
6. Read fiction?
7. Read the newspaper?
8. Read weekly magazines or journals?
9. Attend lectures at a university or other adult education programs?
10. Study (e.g., language learning) at home?
11. Play card games?
12. Play board games (e.g., chess or backgammon)?
13. Play a musical instrument?
14. Go to museums or art exhibitions?
15. Solve crosswords?
16. Solve puzzles, riddles or problems (e.g., Sudoku)?
17. Write letters, e-mail, or other text?
18. Do mathematics (e.g., for work, or for managing household finances)?

Physical Leisure Activities

Try to imagine what a typical summer week looks like for you. How many hours in such a week do you devote to. . .

1. Garden work?
2. Cleaning?
3. Dancing?
4. Walking (e.g., going for walks, to the store, or to the bus)?
5. Riding a bicycle?
6. Strength training?
7. Aerobics?
8. Jogging?
9. Roller skiing?
10. Fishing?
11. Sailing?
12. Hunting?
13. Foraging for mushrooms?
14. Engaging in sport activity (e.g., tennis, golf, or bowling)?
15. Participating in coordination- or flexibility training (e.g., stretching, yoga, or tai chi)?

How physically demanding do you find it to. . .

1. Do garden work?
2. Clean?
3. Dance?
4. Walk (e.g., going for walks, or walking to the bus)?

5. Ride a bicycle?
6. Do strength training?
7. Do aerobics?
8. Go jogging?
9. Go roller skiing?
10. Fish?
11. Sail?
12. Hunt?
13. Forage for mushrooms?
14. Engage in sport activities (e.g., tennis, golf, or bowling)?
15. Participate in coordination- or flexibility training (e.g., stretching, yoga, or tai chi)?

Social Leisure Activities

Try again to imagine what a typical summer week looks like for you. How many hours in such a week do you devote to...

1. Spend time with family members?
2. Talk to family members on the phone?
3. Meet friends?
4. Talk to friends on the phone?
5. Meet relatives?
6. Talk to relatives on the phone?
7. Go with others to a restaurant, pub, or café?
8. Participating in organized community groups (e.g., a soccer club, or a housing association)?
9. Going to parties?
10. Going to church?

S2. Modeling Approach and Prior Specification

We estimated a set of Bayesian univariate and bivariate Latent Change Score Models (LCSMs). Because the data were sparse relative to the number of parameters and covariances in our LCSMs, including multiple exogenous variables in the same model produced parameter shrinkage. To address this, we estimated a separate model for each exogenous variable. For each DRD2 ROI, we thus estimated seven univariate models: one with no exogenous variable, and one each with education, physical activity frequency, physical activity intensity, cognitive activity frequency, cognitive activity intensity, or social activity frequency as the exogenous variable. The same approach was applied to each cognitive variable (working memory, episodic memory, and perceptual speed), yielding 35 models in total.

The latent change estimates displayed in Table 3 of the main text were taken from the models with no exogenous variables; discrepancies across univariate models were minimal. As noted in the main text, bivariate models were estimated for each DRD2 ROI $\times$ cognitive ability combination (six models). Following the same logic, bivariate models were estimated for each leisure activity measure (physical activity frequency, physical activity intensity, cognitive activity frequency, cognitive activity intensity, and social activity frequency) paired with each DRD2 ROI or cognitive ability, yielding 25 additional bivariate models. Finally, 36 bivariate multi-group moderation models were estimated, as described in the main text. Each model was then re-estimated under an alternative set of priors for sensitivity analysis. Including the 9 measurement invariance models and the supplemental covariate and five-activity intensity analyses, a total of 270 models were estimated.

For the factor loadings, the specification of the mean hyperparameter was informed by previous research, including previous COBRA work involving SEM-based analyses (e.g., Köhncke et al., 2018; Nevalainen et al., 2015), leading us to expect that the cognitive indicators will load strongly on their corresponding latent factors. For the correlations, we used a weakly informative beta prior specified as a symmetric distribution centered on zero, representing the assumption that zero or very small correlations are most credible, with monotonically decreasing credibilities for larger coefficient values (for technical discussions on prior specification for covariance matrices, see Depaoli, 2021; Merkle et al., 2021). The remaining priors were weakly informative, and specified to balance vagueness with computational efficiency.

S3. Measurement Invariance

The aim of longitudinal measurement invariance analyses is to examine whether an instrument, such as a cognitive task or a psychometric test, displays sufficiently invariant psychometric properties across

repeated measurements. To test for longitudinal measurement invariance, we specified a baseline longitudinal factor model separately for each cognitive ability, with the T1 indicators for each ability forming a latent T1 factor (and analogously for the T2 indicators), with freely estimated autocorrelations for both the indicators and the two factors in each model. Metric invariance was examined by modifying the baseline models so that the T1 and T2 factor loadings for each cognitive task were constrained to equality between waves, and scalar invariance was examined by additionally constraining the T1 and T2 indicator intercepts to equality between waves. All measurement invariance models utilized the same priors displayed in Table 1 of the main text. The Watanabe–Akaike Information Criterion (WAIC; lower is better; Depaoli, 2021) was used as the model selection criterion. Supplementary Table S1 displays the results of the measurement invariance analyses. For all cognitive abilities, the scalar model showed the lowest WAIC values, suggesting that additional constraints on intercepts did not compromise model fit. The posterior predictive p statistic (PPP) compares the observed data to simulated datasets generated from the posterior distributions of the model parameters. For each simulated dataset and the observed data, a discrepancy statistic is calculated, and the PPP reflects the proportion of posterior predictive discrepancy statistics that exceed those of the observed data. A perfect data-model fit is indicated by a PPP of .50, whereas values below .10 or above .90 suggest poor fit and may warrant model rejection (Garnier-Villarreal & Jorgensen, 2020; Levy, 2011). Inspection of PPP values (Table S1) further suggested that the added constraints did not produce clear model misfits.

Table S1: Longitudinal measurement invariance statistics, model fit indices, and convergence statistics for the cognitive abilities.

Model	PPP	DIC	WAIC	WAIC SE	$B\hat{\Gamma}$	BMc	$\hat{R}$	ESS
WM Configural	0.61	2368.38	2371.79	67.19	0.997	0.995	1.00	5335
WM Metric	0.71	2363.89	2366.74	67.03	0.998	0.997	1.00	4688
WM Scalar	0.66	2362.33	2365.08	66.91	0.998	0.997	1.00	4747
EM Configural	0.34	2313.96	2317.25	66.95	0.990	0.985	1.00	7123
EM Metric	0.36	2312.47	2315.22	67.05	0.991	0.986	1.00	6840
EM Scalar	0.35	2309.71	2312.57	66.74	0.991	0.987	1.00	6565
PS Configural	0.62	1899.69	1902.89	57.36	0.997	0.995	1.00	10867
PS Metric	0.68	1896.30	1899.72	57.38	0.998	0.997	1.00	10983
PS Scalar	0.76	1891.28	1894.63	57.18	0.999	0.998	1.00	7711

Note. WM = Working Memory; EM = Episodic Memory; PS = Perceptual Speed. PPP = Posterior Predictive p-value; DIC = Deviance Information Criterion; WAIC = Watanabe–Akaike Information Criterion; $B\hat{\Gamma}$ = Bayesian Gamma Hat; BMc = Bayesian McDonald's centrality index; $\hat{R}$ = Gelman–Rubin convergence diagnostic (highest across all parameters); ESS = Effective Sample Size (lowest across all parameters).

S4. Model Fit Diagnostics

All 270 estimated models converged successfully (102 main-prior LCSMs, 102 sensitivity-prior LCSMs, 41 supplemental covariate LCSMs, 16 5-activity intensity LCSMs, and 9 measurement invariance CFAs). Across all models, $\hat{R}$ values ranged from 1.000 to 1.004, effective sample sizes (ESS) ranged from 2070 to 9466, and no divergent transitions were observed. Data-model fit was assessed using the posterior predictive p-value (PPP), Bayesian $\hat{\Gamma}$, and Bayesian Mc. The vast majority of models showed good to excellent fit (PPP range: .087–.87; $B\hat{\Gamma}$: .975–1.000; BMc: .919–1.000). One model fell marginally below the conventional PPP $>$ .10 threshold: the caudate–physical intensity bivariate latent change score model estimated in the five-activity intensity sensitivity analysis (Section S7; PPP = .087). Other fit indices for this model remained in the acceptable range ($B\hat{\Gamma}$ = .975, BMc = .962), and we therefore retained it; the corresponding parameter estimates are reported as tentative in the main text. Complete model-by-model fit statistics are available on OSF.

S5. Extended Results

Supplementary Figure S1 displays the estimated correlations between education, leisure activities, and both baseline levels and changes in the three cognitive abilities, as well as the change-change correlations between cognitive changes and leisure activity changes. Supplementary Figure S2 displays the moderation results for all six moderators.

S6. Supplementary Covariate Analyses (Sex and BMI)

To assess whether the main results are robust to potential confounding by sex and body mass index (BMI), we re-estimated all univariate and bivariate LCSMs with sex and BMI included as covariates (regressed on both baseline latent factors and latent change factors). All covariate-controlled models converged (all $\hat{R}$ ¡ 1.003, ESS ¿ 2400, 0 divergences).

The covariate-adjusted results were very similar to the main analyses. The striatal DRD2–cognition change-change correlations remained close to the unadjusted estimates (e.g., putamen–EM: M = .04, 95% HDI [−.02, .11] vs. M = .04 unadjusted; caudate–PS: M = .01, 95% HDI [−.04, .07] vs. M = .03 unadjusted). The longitudinal change estimates were also essentially unchanged after covariate adjustment (caudate DRD2 change: $M = -0.19$; putamen: $M = -0.21$; WM: $M = -0.12$; EM: $M =$

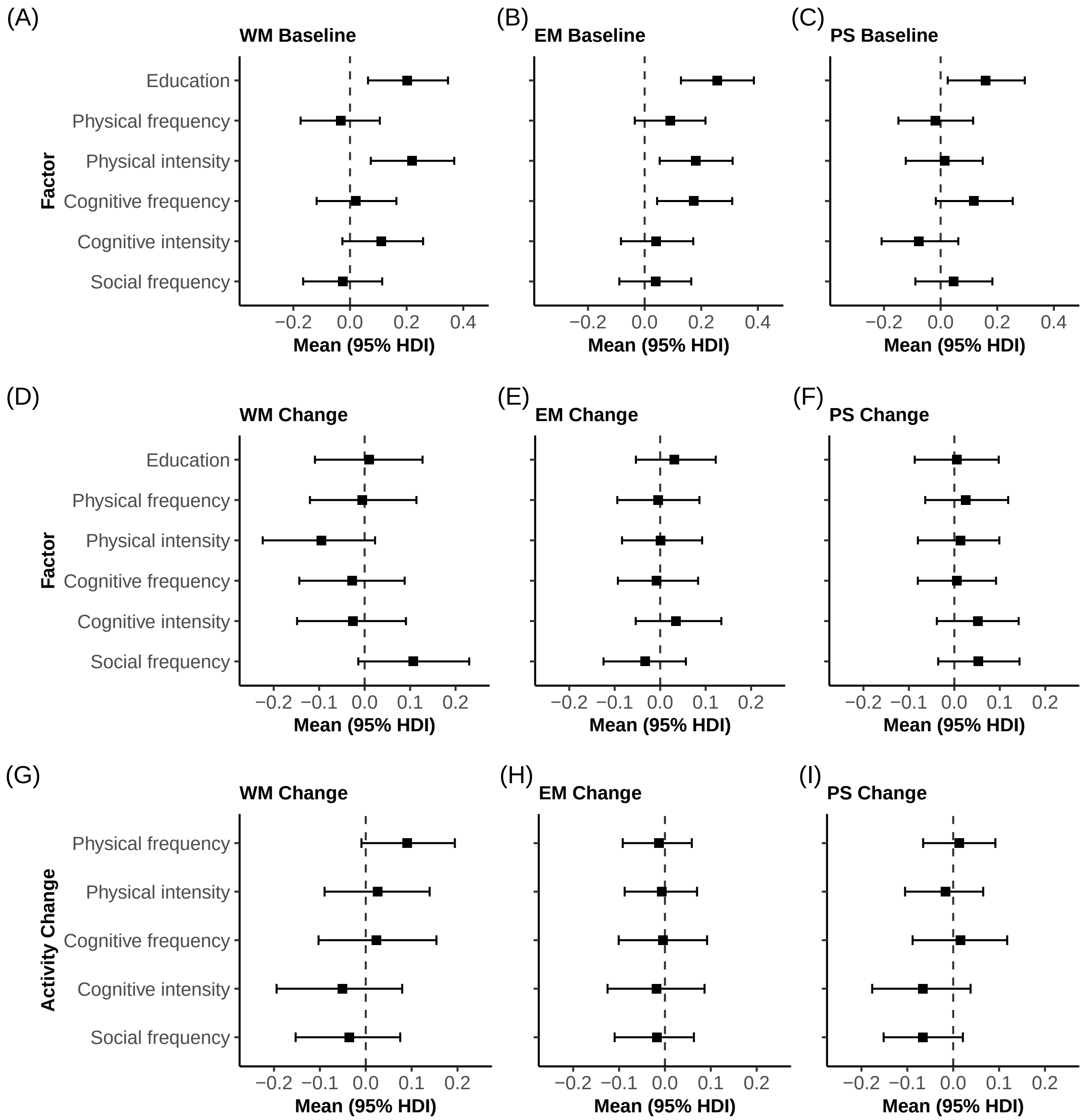

Figure S1: Associations between education, leisure activities, and cognitive abilities. Panels A–C show correlations with baseline cognitive performance; panels D–F show correlations with longitudinal cognitive changes; panels G–I show change-change correlations between cognitive changes and leisure activity changes. Points indicate posterior means, whiskers represent 95% HDIs.

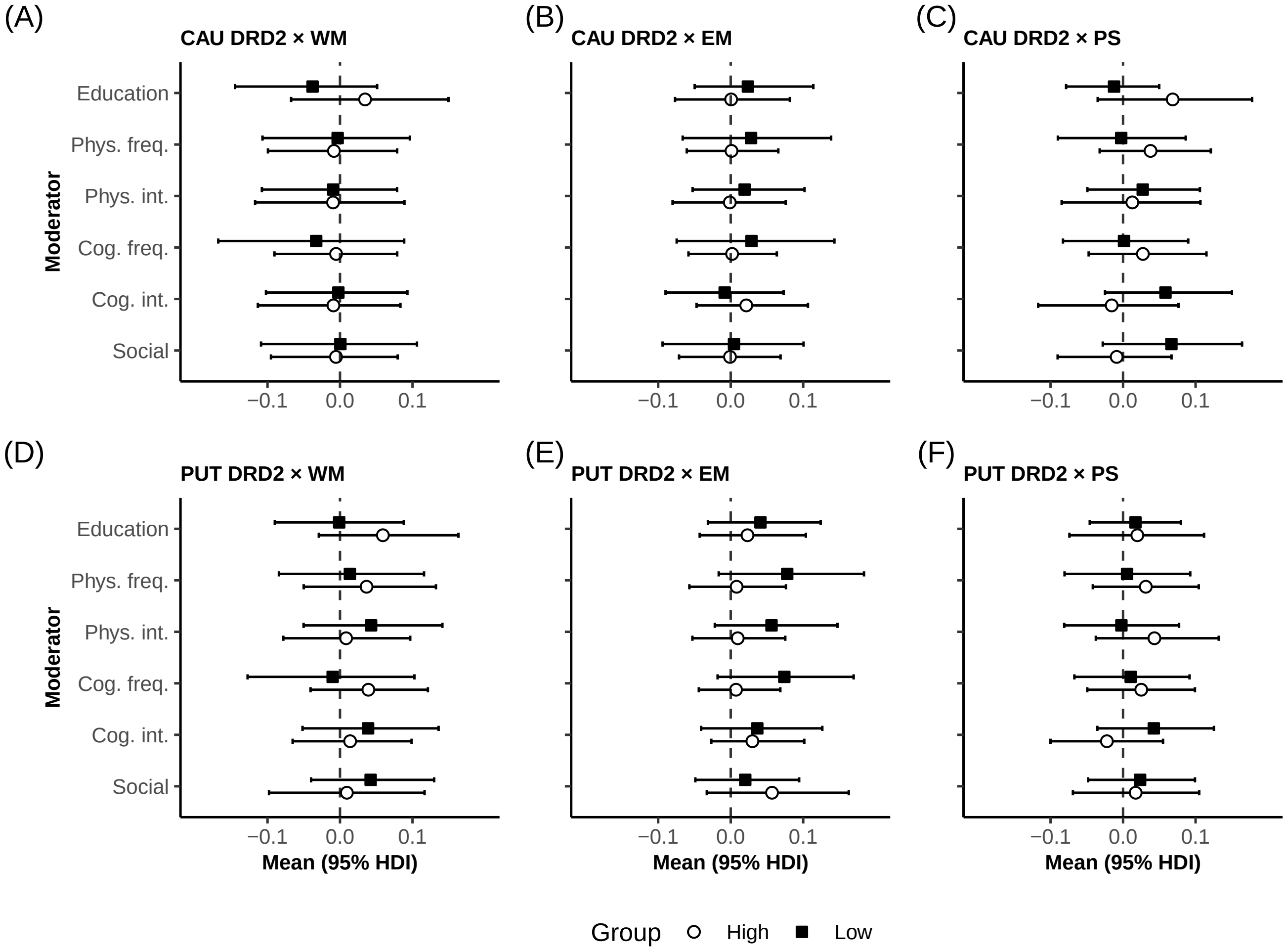

Figure S2: Correlations between changes in striatal DRD2 availability and changes in cognition, estimated separately for the low and high groups of each moderator (median split). Each row corresponds to one striatal ROI; columns show different moderators. Points indicate posterior means, whiskers represent 95% HDIs. Filled squares = low group; open circles = high group.

-0.06; PS: $M = -0.05$). These results indicate that the main findings are not driven by confounding effects of sex or BMI.

S7. Physical Activity Intensity: 5-Activity Subset

As described in the main text, removing the 20% activity engagement cutoff (per reviewer request) expanded the physical activity set from 5 to 15 activities, which introduced low-engagement activities with extreme variance in reported intensity ratings. This changed the composition of the physical activity intensity variable and collapsed the previously observed association between physical activity intensity and baseline striatal DRD2 availability. To evaluate the sensitivity of this finding to the activity composition, we re-estimated all physical intensity models using a restricted intensity variable computed from the 5 most commonly performed physical activities (walking, cycling, jogging, strength training, and sports), following Köhncke et al. (2018). This 5-activity intensity variable has more favorable distributional properties and better represents the intensity of regular physical activity.

Supplementary Table S2 compares the 5-activity intensity results to the all-activity results. With the 5-activity variable, positive associations between physical activity intensity and baseline striatal DRD2 availability were recovered for both the caudate ($M = .12$, $SD = .07$, 95% HDI [$-.01$, .25], $pD_{>0} = .97$) and the putamen ($M = .12$, $SD = .07$, 95% HDI [$-.02$, .27], $pD_{>0} = .95$). The 5-activity associations with baseline cognition were also stronger than the all-activity estimates, particularly for working memory ($M = .25$ vs. .22) and episodic memory ($M = .22$ vs. .18), with 95% HDIs excluding zero in both cases.

The 5-activity change-change correlations between physical intensity changes and DRD2 or cognitive changes were small and uncertain (all 95% HDIs spanning zero), consistent with the main analyses. However, the direction of the putamen DRD2–physical intensity change-change correlation differed between the two variables: the all-activity variable yielded a moderately probable small negative association ($M = -.07$, 95% HDI [$-.18$, .03], $pD_{<0} = .91$), whereas the 5-activity variable yielded a small positive association in the opposite direction ($M = .03$, $pD_{>0} = .69$). This directional flip, together with the fact that neither 95% HDI excludes zero, reinforces our earlier caution that change-change estimates involving physical intensity are sensitive to construct composition and should be interpreted tentatively. The moderation results similarly showed no strong evidence that the 5-activity physical intensity variable moderated the DRD2–cognition change-change association.

These results suggest that the null finding for physical intensity and baseline DRD2 in the main analyses is attributable to the changed construct composition (inclusion of rarely performed activities with

unreliable intensity ratings) rather than a genuine absence of association. When intensity is computed from commonly performed activities, the positive association with baseline striatal DRD2 availability is recovered.

Table S2: Comparison of physical activity intensity associations using all 15 activities versus the 5-activity subset (5 commonly performed activities).

		All activities ($k = 15$)			5-activity subset ($k = 5$)		
Type	Variable	M	95% HDI	$\text{pD}_{>0}$	M	95% HDI	$\text{pD}_{>0}$
Baseline associations							
DRD2	Caudate	.03	[−.11, .16]	.66	.12	[−.01, .25]	.97
DRD2	Putamen	.03	[−.11, .17]	.64	.12	[−.02, .27]	.95
COG	Working memory	.22	[.07, .37]	1.00	.25	[.11, .40]	1.00
COG	Episodic memory	.18	[.05, .31]	1.00	.22	[.10, .36]	1.00
COG	Perceptual speed	.01	[−.13, .15]	.58	.07	[−.06, .21]	.85
DRD2 change associations							
DRD2	Caudate	.03	[−.09, .13]	.68	−.02	[−.13, .09]	.35
DRD2	Putamen	.08	[−.02, .19]	.95	.04	[−.06, .14]	.77
Change-change correlations (DRD2–activity)							
DRD2	Caudate	−.01	[−.12, .11]	.44	.08	[−.07, .25]	.85
DRD2	Putamen	−.07	[−.18, .03]	.09	.03	[−.09, .14]	.69
Change-change correlations (COG–activity)							
COG	Working memory	.03	[−.09, .15]	.69	−.04	[−.18, .09]	.26
COG	Episodic memory	−.01	[−.10, .07]	.35	.02	[−.07, .12]	.68
COG	Perceptual speed	−.01	[−.10, .07]	.39	−.02	[−.12, .08]	.33

Note. M = posterior mean; HDI = highest density interval; $\text{pD}_{>0}$ = probability of direction (proportion of posterior > 0). The all-activity variable includes intensity ratings across all 15 physical activities; the 5-activity variable includes only the 5 most commonly performed activities (walking, cycling, jogging, strength training, sports), following Köhncke et al. (2018).

S8. Sensitivity Analyses: Alternative Priors

The following figures replicate the main analyses and extended results using the alternative (more diffuse) priors specified in Table 1 of the main text. Convergence and fit statistics for the sensitivity models are summarized in Section S4.

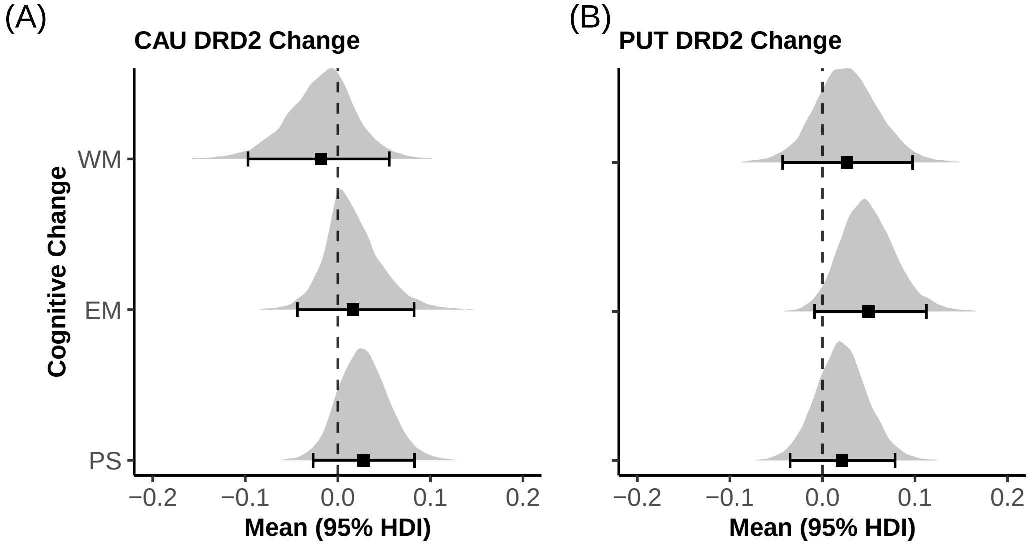

Figure S3: Correlations between changes in striatal DRD2 availability and changes in cognition, estimated using sensitivity priors. This figure replicates the analyses from Figure 2 in the main text. Points indicate posterior means, whiskers represent 95% HDIs, and shaded density plots show posterior distributions.

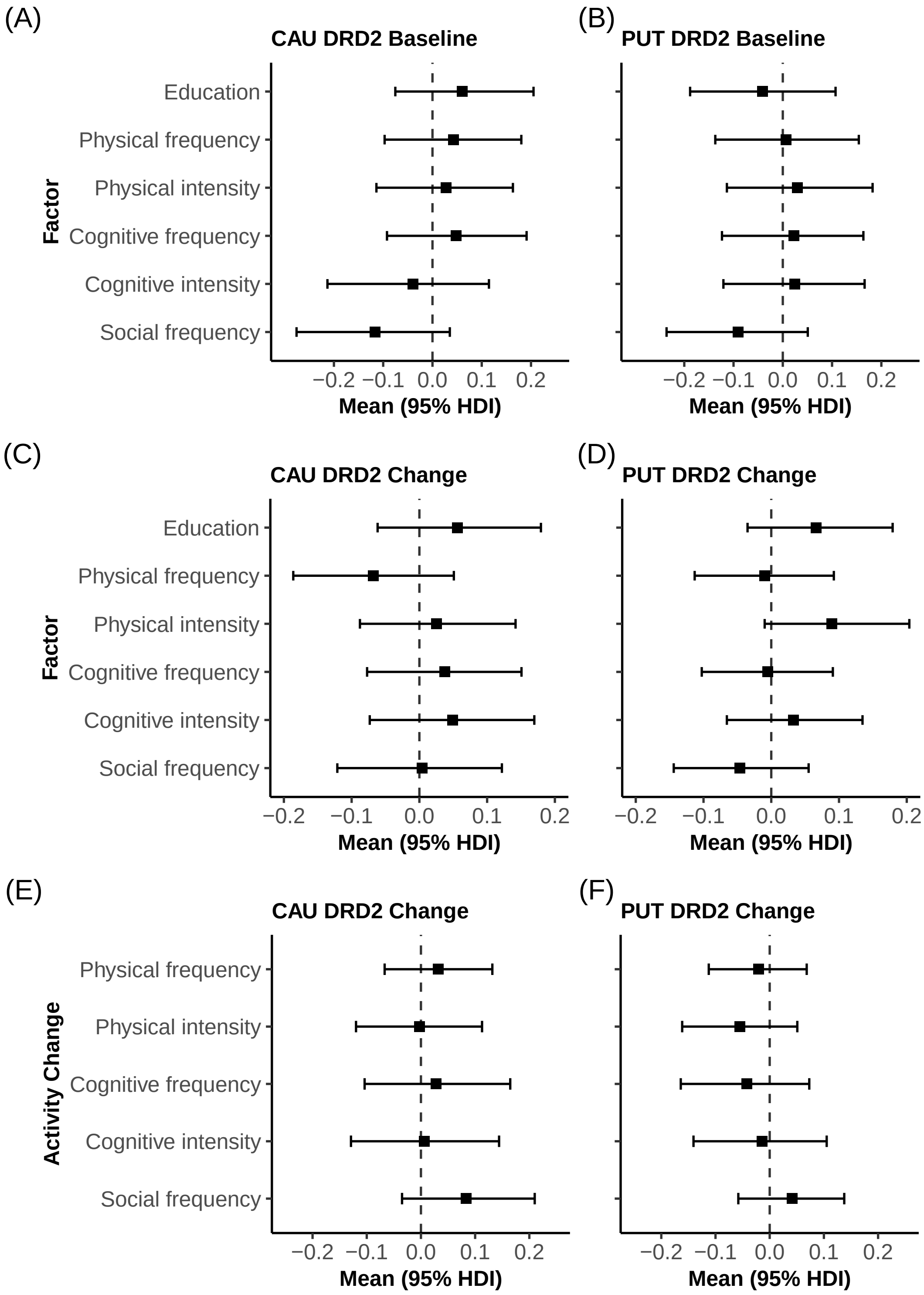

Figure S4: Associations between education, leisure activities, and striatal DRD2 availability, estimated using sensitivity priors. This figure replicates the analyses from Figure 3 in the main text. Panels A–B show correlations with baseline DRD2 levels; panels C–D show correlations with DRD2 changes; panels E–F show change-change correlations between DRD2 changes and leisure activity changes. Points indicate posterior means, whiskers represent 95% HDIs.

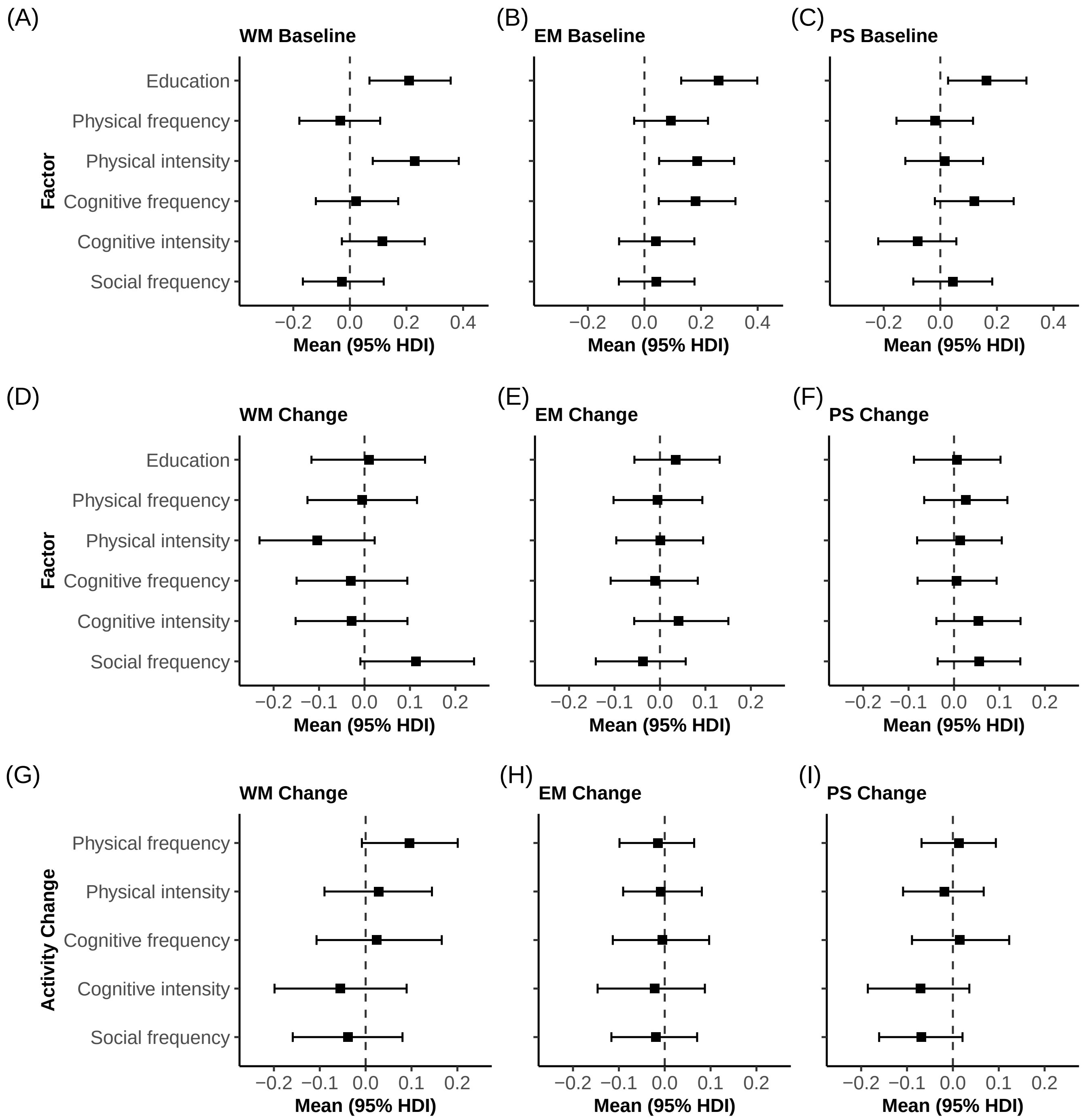

Figure S5: Associations between education, leisure activities, and cognitive abilities, estimated using sensitivity priors. This figure replicates the analyses from Supplementary Figure S1. Panels A–C show correlations with baseline cognitive performance; panels D–F show correlations with cognitive changes; panels G–I show change-change correlations between cognitive changes and leisure activity changes. Points indicate posterior means, whiskers represent 95% HDIs.

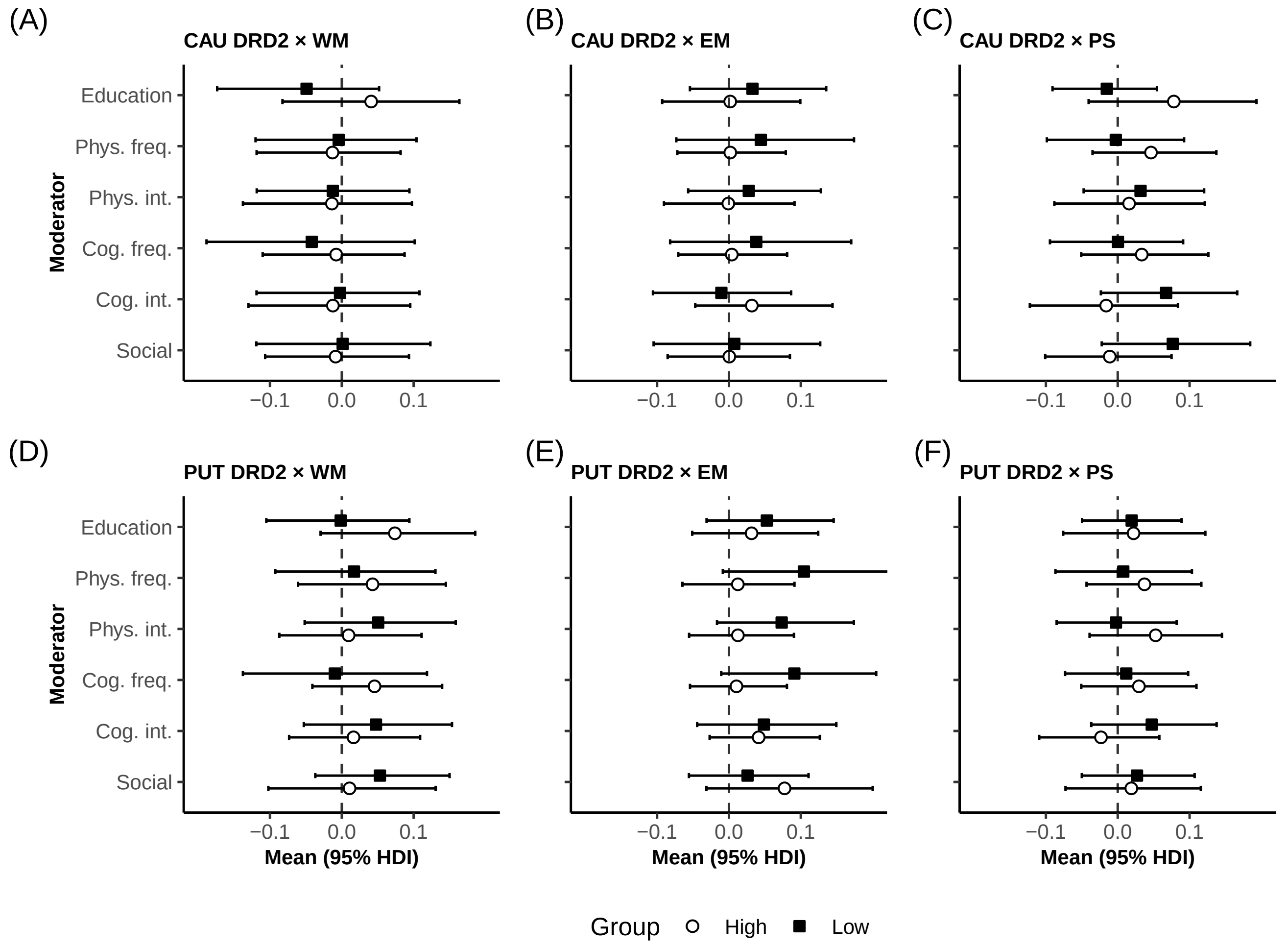

Figure S6: Correlations between changes in striatal DRD2 availability and changes in cognition, estimated separately for the low and high groups of each moderator, using sensitivity priors. This figure replicates the analyses from Supplementary Figure S2. Points indicate posterior means, whiskers represent 95% HDIs.

References

Depaoli, S. (2021). *Bayesian structural equation modeling*. Guilford Press.

Garnier-Villarreal, M., & Jorgensen, T. D. (2020). Adapting fit indices for bayesian structural equation modeling: Comparison to maximum likelihood. *Psychological Methods*, *25*(1), 46–70. https://doi.org/10.1037/met0000224

Köhncke, Y., Papenberg, G., Jonasson, L., Karalija, N., Wåhlin, A., Salami, A., Andersson, M., Axelsson, J. E., Nyberg, L., Riklund, K., Bäckman, L., Lindenberger, U., & Lövdén, M. (2018). Self-rated intensity of habitual physical activities is positively associated with dopamine $D_{2/3}$ receptor availability and cognition. *NeuroImage*, *181*, 605–616. https://doi.org/10.1016/j.neuroimage.2018.07.036

Levy, R. (2011). Bayesian data-model fit assessment for structural equation modeling. *Structural Equation Modeling: A Multidisciplinary Journal*, *18*(4), 663–685. https://doi.org/10.1080/10705511.2011.607723

Merkle, E. C., Fitzsimmons, E., Uanhoro, J., & Goodrich, B. (2021). Efficient Bayesian structural equation modeling in Stan. *Journal of Statistical Software*, *100*(6). https://doi.org/10.18637/jss.v100.i06

Nevalainen, N., Riklund, K., Andersson, M., Axelsson, J., Ögren, M., Lövdén, M., Lindenberger, U., Bäckman, L., & Nyberg, L. (2015). COBRA: A prospective multimodal imaging study of dopamine, brain structure and function, and cognition. *Brain Research*, *1612*, 83–103. https://doi.org/10.1016/j.brainres.2014.09.010
